# A novel x-Ray and γ-Ray combination strategy for radiotherapy after breast-conserving surgery in patients with right breast cancer

**DOI:** 10.3389/fonc.2024.1397273

**Published:** 2024-08-21

**Authors:** Kunpeng Zhang, Ruixin He, Fenwen Tang, Luping Zhou, Xiaozhi Zhang, Jinsheng Li, Zhiwei Wei, Yi Li

**Affiliations:** ^1^ Department of Radiotherapy, The First Affiliated Hospital of Xi’an Jiaotong University, Xi’an, Shaanxi, China; ^2^ Our United Corporation, Xi’an, China

**Keywords:** right breast cancer, radiotherapy after breast-conserving surgery, x-ray and γ-Ray combination strategy, x-ray strategy, dosimetric evaluation

## Abstract

**Background and purpose:**

Radiotherapy is a primary therapeutic approach for breast cancer following breast-conserving surgery. The TaiChiB dual-modality radiotherapy system combining X-ray and focused γ-ray, offers a new approach to reduce the radiation dose of organs at risk (OARs) and has the potential to mitigate the adverse effects of radiotherapy. Currently, there are few studies on the dosimetric characteristics of the TaiChiB dual-modality system for actual treatment plans for specific diseases. The purpose of this work is to study the dosimetric advantages of dual-modal systems for right breast patients after breast-conserving surgery.

**Material and methods:**

Treatment plans for 20 patients with right breast cancer were generated for a linear accelerator (LINAC) based system and the TaiChiB dual-modality system, respectively. Volumetric modulated arc therapy plans with simultaneous integrated boost (VMAT-SIB) were made for the LINAC. Focused γ-ray was used to deliver the boost dose with the dual-modality system. The dosimetric parameters of the target and OARs were evaluated and compared between the treatment plans generated for the two systems.

**Results:**

The TaiChiB dual-modality plans exhibit a higher conformal index (CI) and lower gradient index (GI) for the PGTV and PTV compared with the LINAC-based VMAT-SIB plans. Compared to VMAT-SIB plans, the PTV Dmax, PTV Dmean, PTV V110, PGTV Dmax, and PGTV Dmean of the TaiChiB dual-modality plans are significantly lower. Meanwhile, the dose to OARs, such as the Dmean of the heart, the V5 of liver, the Dmean of ipsilateral lung, the V30 of ipsilateral lung, the V20 of ipsilateral lung, the V5 of ipsilateral lung, the Dmean of contralateral lung, Dmax of contralateral breast and the Dmean of contralateral breast are significantly reduced.

**Conclusions:**

Our study demonstrates the dosimetric advantages of the novel TaiChiB dual-modality radiotherapy system for the treatment of right-sided breast cancer. Overall, for the TaiChiB dual-modality radiotherapy system, the radiation dose outside the target region decreases rapidly, thereby minimizing radiation exposure to neighboring organs and ensuring the conformity of the target area. Our research confirms the potential of the TaiChiB dual-modality system for future radiotherapy.

## 1 Introduction

Breast cancer (BC) is the most frequently diagnosed cancer and the second cause of death by cancer in women worldwide. Its burden has been growing in many parts of the world (1–3). Radiotherapy after breast-conserving surgery can reduce the recurrence rate of tumors in breast tissue and nearby lymph nodes, thereby increasing patient survival rate (4–6). Therefore, radiotherapy is a routine treatment option for most breast cancer patients after breast-conserving surgery. Volumetric modulated arc therapy with a simultaneous integrated boost (VMAT-SIB) technique can deliver more doses to the tumor lesion during the entire breast irradiation period (7–9). VMAT-SIB can form a steep dose gradient and reduce treatment time (10). So, VMAT-SIB is often used for radiotherapy after breast-conserving surgery (11). However, the adverse reactions caused by radiotherapy are still unavoidable. Bartelink H’s research suggests that severe fibrosis is statistically significantly increased as the breast receives higher doses (12). Cheng YJ’s study shows that exposure of the heart to ionizing radiation during radiotherapy for breast cancer increases the subsequent risk of coronary heart disease and cardiac mortality (13). Darby SC demonstrated that the rate of major coronary events increases linearly with the mean dose to the heart by 7.4% per gray (14). In addition, skin toxicity, radiation pneumonitis, hypothyroidism, and contralateral breast cancer are also common adverse reactions of breast cancer radiotherapy (15–17). Minimizing the normal tissue dose and sufficient target dose is the most direct means to reduce the adverse reactions of radiotherapy for breast cancer, and is also the development goal of radiotherapy technology in the past and future.

**Figure 1:**
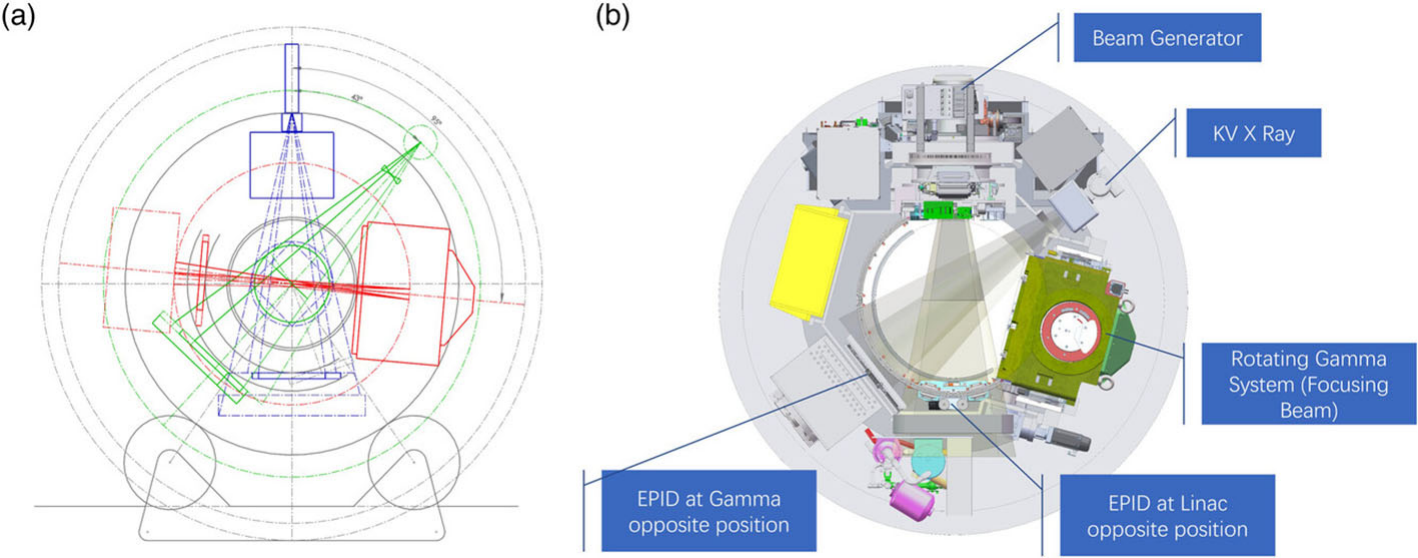
Details of the concept layout and machine profile of TaiChiB system. **(A)** TaiChiB concept layout. Linac, gamma, and kV x-ray are installed in a donut ring gantry with slip ring power supply, and share a same IsoCenter. Blue: 6MV Linac x-ray Beam. Red: Focusing Gamma Beam. Green: KV x-ray Beam; **(B)** TaiChiB machine profile. TaiChiB is a novel teletherapy device combined linear accelerator, rotating gamma radiosurgery system, MV EPID panel and kV image system within an enclosed slip ring gantry, EPID panel has its own track, and can move to linac or gamma opposite position for QA to the above system (20).

VMAT-SIB is usually performed with a linear accelerator (LINAC)-based system for breast cancer patients with breast-conserving surgery (18, 19). As a new system, the TaiChiB dual-modality system combined X-ray, focused γ-rays, and cone-beam CT (CBCT) technology into one integrated radiation therapy system. LINAC-based X-beams and focused γ-beams could be delivered to patients sequentially or simultaneously, as shown in **Figure 1**. The γ-beam system delivers the highest dose to the target’s center and the dose falls off quickly around the 100% prescription dose line (20). This attribute can effectively reduce the normal tissue dose near the target. However, the dose distribution of a treatment plan delivered with the γ-ray system alone may not be good enough to fit a tumor with a large volume or irregular shape. The LINAC-based X-ray system and the focused γ-ray system can be used independently to treat large or small tumors or used together to treat more complicated targets that need a boost dose on part of the volume for a better cure. The TaiChiB dual-modality system was developed to combine the advantages of both independent systems to offer a conformal radiation treatment plan and ensure sufficient dose while minimizing the dose to surrounding normal tissues.

Related studies (4) show that radiation therapy after breast cancer surgery can reduce the incidence of local recurrence. Currently, radiotherapy after breast cancer surgery is performed using X-ray-based linear accelerators (21, 22), with patient-specific radiotherapy plans produced by physicists. However, due to the slow attenuation of X-ray, the dose does not fall quickly enough, which inevitably results in a certain dose to the OARs. At the same time, for treatment areas with tumor lesions, there will be high dose accumulation between the tumor lesions and the PTV, resulting in skin sclerosis. The TaiChiB dual-modality system combines X-rays and γ-ray, providing a new therapeutic approach to radiation therapy. Liu X et al. (20) applied the X‐Ray and γ‐Ray combination strategy to locally advanced pancreatic cancer (LNPC). Their research shows that the strategy is beneficial for local tumor control and the protection of normal organs in patients with LAPC. However, there are currently no studies on the dosimetric advantages of the TaiChiB dual-modality system for right-sided breast cancer radiotherapy planning. The goal of this study is to discover the dose advantage of the TaiChiB dual-modality system in right-sided breast cancer after-surgery radiotherapy planning.

The main innovations and contributions of this research are as follows:

1. This study is the first to use the TaiChiB dual-modality system to produce a larger number of right breast radiotherapy plans.
2. We compared the quality of the treatment plans made for the TaiChiB dual-modality system and other systems (such as VMAT-SIB).
3. This study strongly demonstrates the dosimetric advantages of the TaiChiB dual-modality system for right breast radiotherapy through statistical analysis.

## 2 Materials and methods

### 2.1 Patient selection

This retrospective analysis involved 20 individuals diagnosed with right breast cancer who received Linac-based VMAT-SIB treatment at the Radiotherapy Department of XXX from June 2022 to June 2023. The patients were chosen randomly from the institution.

### 2.2 Patient positioning

All individuals were immobilized in the supine position using a breast bracket, with their hands raised, during CT simulation. Every participant underwent a helical CT scan while breathing freely using a 16-slice CT scanner (Big bore, Philips Medical Systems, Cleveland, OH). The scanning parameters comprised a pixel spacing of 1.1543mm×1.1543mm, a matrix of 512×512, a pitch of 0.85, 120KV, 400mAs, a thickness of 5 mm, and a layer spacing of 5 mm. Subsequently, the scanned images were transferred to a treatment planning system (Monaco Version 5.11.02, Elekta, Sweden).

### 2.3 TaiChiB dual-modality system

#### 2.3.1 System specifications and basic features

Currently, the TAICHI device used in clinical settings is a multi-modal integrated radiotherapy system developed and manufactured by Xi’an Da’an Gene Group Co., Ltd. The TAICHI treatment head consists of two main components: the first is a linear accelerator with a non-flattening filter mode of 6MV radiation energy. Inside the accelerator head, there is a pair of lead doors and a multi-leaf collimator containing 60 pairs of leaves. The multi-leaf collimator is composed of 40 pairs of thin leaves with a thickness of 0.5cm in the middle and 20 pairs of thick leaves with a thickness of 1cm on the sides. Additionally, the accelerator treatment head can rotate at a speed of 6°/s and deliver a continuously variable dose rate of up to 1400cGy/min for treatment. The second component is a gamma-focusing head equipped with 18 sources and 7 sets of collimators, which treat the lesion through multi-source focusing. TAICHI has upgraded the traditional gamma knife point irradiation to continuous arc beam therapy. Furthermore, the gamma head itself can rotate to achieve non-coplanar irradiation from 0° to -14°, better protecting critical organs adjacent to the target area in clinical settings.

The TAICHI device can combine the dual characteristics of the gamma knife and accelerator head, fully utilizing the physical advantage of the gamma knife in the dose drop-off rate. By increasing the central dose of the target area and using gamma knife irradiation for the boost area, the X/γ combined irradiation planning method is employed to optimize the dose distribution.

For plans requiring multiple arc pulls, TAICHI can achieve uninterrupted arc pulling. Unlike traditional C-arm accelerators, the TAICHI gantry is mounted on a slip ring structure, allowing for treatment arcs without angle restrictions. For tumors in the back, TAICHI can cross 180° for beam delivery, enhancing planning modulation capabilities and treatment efficiency. We summarize the different parameters of the Dual-modality system and C-type Linac as shown in **Table 1**.

**Table 1:** The parameters of the TaiChiB dual-modality system and C-type Linac.

Technology	Dual-modality system	C-type Linac
Field angle	Unlimited	The arc angle is limited to 180°
Target dose	When irradiating a large target area, the dose of the local target area is greatly increased.	When irradiating a large target area, the incremental dose to the local target area is limited.
Organ-risk dose	Due to the higher dose gradient, high doses are delivered to the target area while reducing doses to surrounding organs at risk.	The boost target area will relatively increase the dose to surrounding organs at risk under conventional segmentation conditions.
Stability	Slip ring structure has a longer service life	When the accelerator rack is used for a long time, the cantilever will be deformed.

#### 2.3.2 The production process of the TaiChiB dual-modality plan

Firstly, the physicist needs to design a Gamma Knife plan for the target volume to be treated. After the Gamma Knife plan is completed, it needs to be submitted to a senior physicist for plan review. Once the review is passed, a new accelerator plan is created, and in the optimization interface, the previously completed Gamma Knife plan dose is used as the base dose to optimize the accelerator. Finally, the production of the combined Gamma Knife and accelerator plan is completed and submitted to a senior physicist for plan review.

### 2.4 Treatment planning

The region of the affected breast that extends 1.5cm outward from the silver clip following surgery is referred to as the gross tumor volume (GTV). The planning gross tumor volume (PGTV) is defined as the GTV with an additional 0.5 cm margin. The clinical target volume (CTV) encompasses the entire affected breast, and in cases where the patient is N1, it also includes the supraclavicular and inferior lymphatic drainage areas. All borders of the CTV are expanded by 0.5cm to create the planning target volume (PTV), except the anterior border. The expansion of the posterior border does not include lung tissue.

#### 2.4.1 VMAT planning

The VMAT-SIB plans were generated using the Monaco treatment planning system (Elekta, Inc. Sweden. Version: 5.11.02). The plans consisted of two 220° partial arcs with 150 control points, and the dose calculation grid was 3mm. The prescribed dose for 25 treatment fractions was 50 Gy for the PTV and 60 Gy for the PGTV. The OARs encompassed the heart, ipsilateral lung, contralateral lung, total lung, contralateral breast, liver, and spinal cord prv. The dose calculation algorithm employed was the Monte Carlo (XVMC) method.

#### 2.4.2 TAICHIB planning

The RayStation treatment planning system (RT Pro TPS V2) was employed to produce the TaichiB treatment plan. Focused γ-ray beams were utilized to administer an additional dose to the PGTV, while the optimization of the Linac treatment plan was carried out to encompass the necessary boost dose for PTV coverage. The prescription dose for each case was consistent with that of the VMAT-SIB plan. A comparison was made between the TaichiB plans and the VMAT-SIB plans. To ensure plan quality and uniformity, all TaichiB plans were developed by a proficient medical physicist. The dose calculation algorithm utilized in the TaichiB plan in this investigation is the collapsed cone convolution method.

### 2.5 Assessment method

The assessment parameters for the prescribed dose in the target volume encompass the maximum dose (Dmax), the volume of the target area receiving 110% of the prescribed dose (V110), the conformity index (CI), and the gradient index (GI). The CI is calculated as (Vt_ref/Vt) × (Vt_ref/Vref), where Vt represents the volume of the target area, Vt_ref denotes the volume of the target receiving a dose equal to or higher than the reference dose, and Vref signifies the total volume receiving a dose equal to or higher than the reference dose (23). The CI ranges from 0 to 1, a higher value indicating greater conformality. The GI is defined as the ratio of the volume receiving 50% of the prescribed dose or more to the volume receiving 100% of the prescribed dose or more (20). To calculate the GI of PGTV, the PTV prescription dose (50Gy) needs to be subtracted. The GI characterizes the steepness of the dose gradient, with a lower GI value indicating a more rapid dose falloff (24).

The OARs under evaluation include the heart, ipsilateral lung, contralateral lung, lungs, contralateral breast, liver, and spinal cord prv. The evaluation criteria encompass the maximum dose (Dmax) of the spinal cord prv, the mean dose (Dmean) and volume receiving 5 Gy (V5) of the heart, the Dmean, V20, and V5 of the ipsilateral lung, contralateral lung, and lungs, the Dmean of the contralateral breast, and the V5 of the liver.

The data processing was conducted using SPSS-19 statistical software, and the data were presented in the form of mean ± standard deviation. Paired t-tests were utilized to analyze the two sets of calculations, with the significance of the difference indicated by P values (<0.05).

## 3 Results

**Figure 2:**
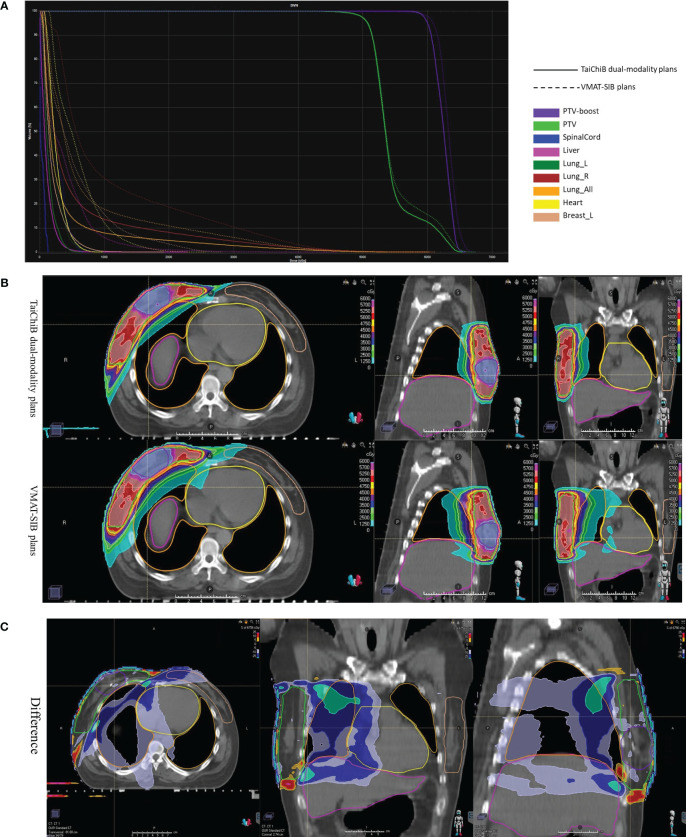
DVH **(A)** and dose distribution **(B)** were analyzed for a patient undergoing treatment with TaiChi dual-modality plans and VMAT-SIB plans. The difference in the dose distributions (TaiChi dual-modality-VMAT-SIB) was illustrated in **(C)**.

A comparative analysis of treatment planning was conducted for 20 patients with right breast cancer, comparing the techniques of VMAT-SIB and TaiChiB. **Figure 2** displayed the dose volume histograms (DVH) (a) and dose distributions (b) for one of the patients, illustrating the results of the TaiChiB and VMAT-SIB treatment plans. Meanwhile, the difference in dose distribution between TaiChiB and VMAT-SIB plans is also shown in (c). The CI and the GI of the PGTV for each case were displayed in **Table 2**, whereas the CI and GI of the PTV were presented in **Table 3**. The CIs for the PTV (p=0.004) or the PGTV (p=0.013) show significant difference when comparing TaiChiB plans with VMAT-SIB plans. The TaiChiB plans exhibit a higher CI value of 0.85 ± 0.06 versus 0.77 ± 0.15 for the PGTV and 0.86 ± 0.04 versus 0.84 ± 0.05 for the PTV. In addition, The GI for the PTV (p=0.000) or the PGTV (p=0.000) also shows significant difference between the two kinds of plans. The TaiChiB plans have a lower GI value of 2.81 ± 2.62 versus 5.94 ± 3.76 for the PGTV and 2.20 ± 0.28 versus 2.50 ± 0.39 for the PTV.

**Table 2:** Values of the CI and GI of the PGTV.

PGTV	CI		GI	
	VMAT-SIB PLAN	TAICHIB PLAN	VMAT-SIB PLAN	TAICHIB PLAN
Case1	1.30	0.87	3.29	1.67
Case2	0.70	0.84	11.43	2.10
Case3	0.83	0.87	3.58	2.23
Case4	0.58	0.68	9.25	13.54
Case5	0.56	0.70	11.74	2.93
Case6	0.76	0.89	7.53	1.82
Case7	0.68	0.77	7.51	2.59
Case8	0.75	0.84	5.05	1.90
Case9	0.76	0.84	15.11	3.92
Case10	0.75	0.80	7.46	4.14
Case11	0.69	0.88	9.21	1.87
Case12	0.80	0.88	3.72	1.95
Case13	0.73	0.88	2.55	1.77
Case14	0.76	0.90	2.33	2.05
Case15	0.71	0.85	3.28	2.19
Case16	0.83	0.89	2.49	1.82
Case17	0.70	0.88	4.21	2.11
Case18	0.84	0.85	3.92	2.63
Case19	0.85	0.91	2.29	1.54
Case20	0.80	0.92	2.89	1.47
Mean	0.77 ± 0.15	0.85 ± 0.06	5.94 ± 3.76	2.81 ± 2.62
P-value	0.013		0.001	

**Table 3:** Values of the CI and GI of the PTV.

PTV	CI		GI	
	VMAT-SIB PLAN	TAICHIB PLAN	VMAT-SIB PLAN	TAICHIB PLAN
Case1	0.81	0.84	2.58	2.15
Case2	0.79	0.89	2.43	2.44
Case3	0.79	0.81	2.38	2.00
Case4	0.82	0.82	2.67	2.43
Case5	0.72	0.79	3.75	2.87
Case6	0.87	0.90	2.54	2.24
Case7	0.86	0.85	2.76	2.60
Case8	0.83	0.87	2.20	2.00
Case9	0.88	0.90	2.39	2.20
Case10	0.71	0.73	1.94	1.71
Case11	0.81	0.84	2.76	2.42
Case12	0.86	0.87	2.40	2.16
Case13	0.87	0.87	2.15	1.88
Case14	0.87	0.87	2.52	2.07
Case15	0.86	0.89	2.80	2.22
Case16	0.87	0.86	2.21	1.97
Case17	0.87	0.89	2.36	2.23
Case18	0.85	0.85	2.89	2.42
Case19	0.88	0.89	2.04	1.84
Case20	0.91	0.91	2.25	2.05
Mean	0.84 ± 0.05	0.86 ± 0.04	2.50 ± 0.39	2.20 ± 0.28
P-value	0.004		0.000	

The dosimetric parameters about the targets are documented in **Table 4**. The TaiChiB plans show significant decreases in the Dmax of PTV (5335.8 ± 74.622 versus 5445.6 ± 96.852, p=0.000), the Dmax of PGTV (6593.30 ± 68.41 versus 6707.20 ± 111.42, p=0.002), the Dmean of PTV (5348.10 ± 65.74 versus 5455.75 ± 86.24, p=0.000), the Dmean of PGTV (6256.50 ± 37.77 versus 6291.20 ± 33.52, p=0.015) and the V110% PTV (14.71 ± 6.48 versus 34.48 ± 14.19, p = 0.000) comparing with the VMAT-SIB plans. Then, the Dmin of PGTV for the TaiChiB plans (5387.60 ± 325.23 versus 5179.05 ± 361.60, p=0.018) shows a significant improvement compared to the VMAT-SIB plans.

**Table 4:** Dosimetric parameters for the target region between VMAT-SIB plans and TaiChiB dual-modality plans.

Target volume	Parameter	VMAT-SIB	TAICHIB	T value	P value
PGTV	Dmax/Gy Dmin/Gy Dmean/Gy V_110_/%	6707.20 ± 111.42 5179.05 ± 361.60 6291.20 ± 33.524 1.85 ± 2.17	6593.30 ± 68.41 5387.60 ± 325.23 6256.50 ± 37.77 1.31 ± 5.34	3.687 -2.589 2.683 0.385	0.002 0.018 0.015 0.705
PTV	Dmax/Gy Dmin/Gy Dmean/Gy V_110_/%	6707.20 ± 111.42 3538.35 ± 411.10 5455.75 ± 86.24 34.48 ± 14.19	6593.30 ± 68.41 3573.95 ± 500.91 5348.10 ± 65.74 14.71 ± 6.48	3.687 -0.397 7.665 5.885	0.002 0.696 0.000 0.000

The dosimetric parameters for organs at risk (OARs) are outlined in **Table 5**. The TaiChiB treatment plans demonstrated their effectiveness in minimizing the radiation dose to the OARs. In comparison to the VMAT-SIB plans, the TaiChiB plans show significant lower Dmean (973.60 ± 273.72 versus 1282.50 ± 279.04, p=0.000), V30 (11.02 ± 4.23 versus 15.74 ± 4.95, p=0.000), V20 (16.62 ± 5.65 versus 23.00 ± 6.52, p=0.000), V10 (25.18 ± 7.98 versus 35.67 ± 8.69, p=0.000) and V5 (39.58 ± 11.05 versus 54.74 ± 10.62, p=0.000) values for the ipsilateral lung. In addition, the Dmean of the heart (273.15 ± 127.16 versus 395.05 ± 124.81, p=0.000), contralateral lung (194.25 ± 83.22 versus 268.70 ± 60.31, p=0.000), and contralateral breast (336.70 ± 141.88 versus 426.60 ± 156.99, p=0.000) of the TaiChiB plans are also significantly lower. Furthermore, there was a significant reduction in the V5 of liver (11.43 ± 6.70 versus 17.15 ± 9.78, p=0.022), the V5 of lungs (23.74 ± 7.64 versus 33.60 ± 6.75, p=0.000), the V5 of contralateral lung (5.82 ± 5.23 versus 8.05 ± 6.06, p=0.043), the V10 of heart (1.57 ± 3.70 versus 3.55 ± 5.56, p=0.044) and the Dmax of contralateral breast (2271.8 ± 826.0 versus 2848.55 ± 860.8, p=0.002) for the TaiChiB plans.

**Table 5:** Dosimetric parameters for organs at risk between VMAT-SIB plans and TaiChiB dual-modality plans.

OARs	Parameter	VMAT	TAICHIB	T value	P value
Spinalcord prv	Dmax/Gy	1009.75 ± 791.96	975.25 ± 1040.92	0.407	0.689
Heart	Dmean/Gy	395.05 ± 124.81	273.15 ± 127.16	4.623	0.000
Heart	Dmax/Gy	1683.3 ± 826.9	1396.7 ± 894.1	1.924	0.070
Heart	V10/%	3.55 ± 5.56	1.57 ± 3.70	2.155	0.044
Heart	V5/%	21.54 ± 16.71	29.29 ± 79.11	0.415	0.683
Ipsilateral lung	Dmean/Gy	1282.50 ± 279.04	973.60 ± 273.72	11.90	0.000
Ipsilateral lung	V30/%	15.74 ± 4.95	11.02 ± 4.23	10.142	0.000
Ipsilateral lung	V20/%	23.00 ± 6.52	16.62 ± 5.65	8.326	0.000
Ipsilateral lung	V10/%	35.67 ± 8.69	25.18 ± 7.98	9.560	0.000
Ipsilateral lung	V5/%	54.74 ± 10.62	39.58 ± 11.05	9.816	0.000
contralateral lung	Dmean/Gy	268.70 ± 60.31	194.25 ± 83.22	5.716	0.000
contralateral lung	V20/%	0.004 ± 0.01	0.04 ± 0.14	1.189	0.249
contralateral lung	V5/%	8.05 ± 6.06	5.82 ± 5.23	2.169	0.043
Lungs	Dmean/Gy	834.20 ± 166.83	598.50 ± 220.85	6.594	0.000
Lungs	V20/%	12.83 ± 3.63	9.26 ± 3.14	8.104	0.000
Lungs	V5/%	33.60 ± 6.75	23.74 ± 7.64	10.043	0.000
contralateral breast	Dmean/Gy	426.60 ± 156.99	336.70 ± 141.88	4.488	0.000
contralateral breast	Dmax/Gy	2848.55 860.8	2271.8 826.0	3.509	0.002
Liver	V5/%	17.15 ± 9.78	11.43 ± 6.70	2.498	0.022

## 4 Discussion

Radiation therapy (RT) is an essential component of the therapeutic approach for breast cancer. Research has demonstrated that the utilization of radiotherapy in breast cancer patients can significantly decrease the likelihood of local recurrence and metastasis within 5 years (25). Before this research, the LINAC-based VMAT-SIB treatment had been commonly utilized in clinical practice, demonstrating favorable dose distribution and beneficial treatment outcomes for patients diagnosed with breast cancer. As the prescribed dose for LINAC-based VMAT-SIB treatment rises, there is a corresponding increase in the occurrence of radiation-induced skin toxicity within the treated area, with varying degrees of severity. Additionally, there is a potential for concurrent elevation of radiation-induced pneumonitis and ischemic heart disease. The innovative TaiChiB dual-modality radiation system was developed as a potential novel solution to reduce the probability of the aforementioned issue occurring in this particular field. The focused γ-beam system can administer the maximum dose to the target while exhibiting a rapid dose reduction outside the target area. The LINAC system is capable of delivering highly effective treatment for large targets. As a result, the TaiChiB dual-modality system integrates the advantages of two distinct systems to produce a treatment plan that is better tailored to the target area and minimizes radiation exposure to organs at risk. Due to the rapid drop in dose, for treatment areas with tumor lesions, there is low dose accumulation between the tumor lesions and the PTV.

Our research involves the selection of 20 patients diagnosed with right breast cancer, and subsequently compare the treatment plans produced by the LINAC-based VMAT systems. The dosimetric parameters of the target area are evaluated based on the critical importance of conformity index (CI) as a key quality metric for radiation treatment plans. **Table 6** summarizes the target CI values of multiple breast cancer radiotherapy planning studies. In the research on right breast cancer conducted by Liu Y-C and colleagues (26), the CI values for the PTV in IMRT, hybrid 3D-CRT/IMRT, non-continuous partial arc, and continuous partial arc radiation therapy plans were 0.64 ± 0.05, 0.68 ± 0.03, 0.74 ± 0.01, and 0.74 ± 0.01, respectively. In Wei S. et al’s study, 50 patients with left breast cancer were considered, and the CI value of PTV in IMRT plans was 0.67 ± 0.07 (27). In a study on right breast cancer by Suyan B. et al, the CI value for PTV in IMRT, H-IMRT, and H-VMAT plans were reported as 0.63 ± 0.08, 0.65 ± 0.1, and 0.65 ± 0.09, respectively (28). In our research, The CI values for the PGTV and PTV of the TaiChiB plans are 0.85 ± 0.06 and 0.86 ± 0.04, respectively, which is significantly higher than the LINAC-based VMAT-SIB plans. The LINAC-based VMAT-SIB plans prioritize minimizing the dosage of organs at risk (OARs) at the expense of target area conformity. Innovatively, the TaiChiB system integrates the benefits of both focused γ-rays and X-rays to address this issue. The initial treatment plan is developed using the focused γ-beam system and is later enhanced with LINAC-based x-ray treatment technology. As a result, the TaiChiB dual-modality system produces a plan that is better tailored to the target area. Significantly, the Dmean value of PGTV, the Dmean value of PTV, the Dmax value of PGTV, and the Dmax value of PTV are observed to be lower in comparison to that of the VMAT system group. The reduced values of Dmean and Dmax suggest that the plan implemented by the TaiChiB system results in fewer hot spots and more uniform dose distribution. The TaiChiB dual-modality system exhibits significant attenuation characteristics for γ-rays, resulting in a rapid decrease in deposited energy from γ-rays outside the 100% prescription dose line. It is particularly significant to note that the majority of breast cancer dose comparison studies do not include an assessment of the gastrointestinal (GI) parameter. Our research findings indicate that the GI value of the TaiChiB treatment plan is significantly lower compared to the VMAT-SIB treatment plan, suggesting a steeper gradient and faster dose drop.

**Table 6:** The Comparison of CI between different studies.

Liu Y-C.et al (24)	Continuous Partial Arc	0.74 ± 0.01
	Non-Continuous Partial Arc	0.74 ± 0.01
	Hybrid 3D-CRT/IMRT	0.68 ± 0.03
	IMRT	0.64 ± 0.05
Wei S. et al. (25)	IMRT	0.67 ± 0.07
Suyan B. et al. (26)	IMRT	0.63 ± 0.08
	H-IMRT	0.65 ± 0.10
	H-VMAT	0.650 ± 0.09
Our research	VMAT-SIB	0.77 ± 0.15 (PGTV)
		0.84 ± 0.05 (PTV)
	TAICHIB	0.85 ± 0.06 (PGTV)
		0.86 ± 0.04 (PTV)

The most concerning aspect about OARs is the level of radiation exposure to the ipsilateral lung, contralateral breast, and heart. In our research, the values of Dmean, V_30_, V_20_, V_10,_ and V_5_ of the ipsilateral lung in the SIB-VMAT plans are 1282.50 ± 279.04, 15.74 ± 4.95, 23.00 ± 6.52, 35.67 ± 8.69 and 54.74 ± 10.62, respectively. The values of Dmean, V_30_, V_20_, V_10,_ and V_5_ of the ipsilateral lung in the TaiChiB plans are 973.60 ± 273.72, 11.02 ± 4.23, 16.62 ± 5.65, 25.18 ± 7.98 and 39.58 ± 11.05, respectively. In comparison to the SIB-VMAT plans, the TaiChiB plans demonstrate a statistically significant reduction in the irradiation dose to the ipsilateral lungs. In the research conducted by Wei S et al. (27), the Dmean, V_30_, V_20_, V_10,_ and V_5_ in the ipsilateral lung of the IMRT plans exhibit values of 1330 ± 65, 16.34 ± 1.71, 22.49 ± 1.37, 36.87 ± 2.17 and 59.27 ± 4.24, respectively, which are still higher than those of the TaiChiB plans. In the investigation of the right breast, Bi S et al. (28), prescribed doses of 49.5 Gy for the PGTV and 43.5 Gy for the PTV. Despite this, the VMAT plan that was implemented doesn’t result in lower ipsilateral lungs (V5, 41.50 ± 9.97) dose compared to the the TaiChiB plans. In our research, the Dmean of contralateral breast in the SIB-VMAT plans and TaiChiB plans were 426.60 ± 156.99 and 336.70 ± 141.88, respectively, with a statistically significant p-value of 0.000. The V_5_ of contralateral breast in the SIB-VMAT plans and TaiChiB plans were 8.05 ± 6.06 and 5.82 ± 5.23, respectively, with a statistically significant p-value of 0.043. Furthermore, in comparison to the SIB-VMAT plans, there is a significant reduction in the radiation dose delivered to the lungs. In addition, compared to SIB-VMAT plans, the Dmean of the heart (1.57 ± 3.70 versus 3.55 ± 5.56) and the V_10_ of the heart (1.57 ± 3.70 versus 3.55 ± 5.56) are significantly reduced with p-values 0.000 and 0.044 respectively. It is also worth noting that the Dmax of the contralateral breast and the V5 of the liver of TaiChiB plans is significantly lower than SIB-VMAT plans. The experimental findings demonstrate that the TaiChiB system effectively minimizes radiation exposure to OARs to a significant degree. The excellent performance is attributed to the dual-modality of the TaiChiB system, which enables the delivery of a high dose to the center of the target area while rapidly decreasing around the 100% isodose line.

In our research, the TaiChiB dual-modality system represents a unique clinical modality. This equipment integrates focused γ- rays and X-rays for the first time. Our study is the inaugural assessment of the efficacy of the dual-modality system’s radiotherapy plan for right breast cancer radiotherapy. The research confirms that this system can offer superior clinical plan quality and serve as a reference for further investigation of the TaiChiB dual-modality system in other diseases. Nevertheless, there are several unresolved issues in this study, such as patient sample size, the absence of plan validation in the phantom, TCP, NTCP, and patient follow-up results. At present, the number of cases involved in the comparison is only 20 and it is meaningful for planning dosimetric comparisons. However, there is still a need to increase the amount of data to enhance the persuasiveness of the study and general clinical acceptability. Plan phantom verification is a measure of the deviation between the treatment plan and the actual output of the machine. Plan verification based on γ- rays and X-rays is still in the development stage, so there are currently no conditions to provide it. TCP and NTCP are biological reactions and are not the focus of our current research. The TaiChiB dual-modality system is a new technology that lacks clinical application accumulation and does not have the conditions for follow-up after radiotherapy.

Our current study is based only on the dosimetry of breast cancer on the right side, and there is a lack of evaluation of biological effects and clinical follow-up of patients with radiation response after treatment. In the future, we will focus on exploring the biological effects of multimodal systems on breast cancer and increasing the statistics of radiation response in patients after clinical radiotherapy. At the same time, we will continue to explore the advantages of the TaiChiB dual-modality system in radiotherapy for other cancer types.

## 5 Conclusion

Our research presents a novel and improved approach for radiotherapy in patients with right-sided breast cancer. The dual-modal system combines the use of γ-beam and X-ray. When the prescribed dose is reached within the target area, the radiation dose outside the target region decreases rapidly, thereby minimizing radiation exposure to neighboring organs and ensuring the conformity of the target area. This research confirms the potential of the TaiChiB dual-modality system for future radiation therapy.

## Data Availability

The original contributions presented in the study are included in the article/supplementary material. Further inquiries can be directed to the corresponding author.
